# Age, motor dysfunction and neuropsychiatric symptoms impact quality of life in multiple sclerosis

**DOI:** 10.1590/0034-7167-2021-0207

**Published:** 2022-06-06

**Authors:** Patrícia de Morais Ferreira Brandão, Tayla Borges Lino, Renata Terra de Oliveira, Andrelisa Vendrami Parra, Paulo Henrique Muleta Andrade, Gustavo Christofoletti

**Affiliations:** I Universidade Federal de Mato Grosso do Sul. Campo Grande, Mato Grosso do Sul, Brazil.; II APAE de Campo Grande, Centro Especializado de Reabilitação. Campo Grande, Mato Grosso do Sul, Brazil.

**Keywords:** Multiple Sclerosis, Quality of Life, Severity of Illness Index, Age Factors, Regression Analysis, Esclerose Múltipla, Qualidade de Vida, Índice de Gravidade da Doença, Fatores Etários, Análise de Regressão, Esclerose Múltiple, Calidad de Vida, Índice de Severidad de la Enfermedad, Factores de Edad, Análisis de Regresión

## Abstract

**Objectives:**

to investigate the impact of age, motor dysfunction and neuropsychiatric symptoms on the quality of life of people with multiple sclerosis in comparison to healthy peers.

**Methods:**

a total of 141 participants were tested in a single session. The assessments were composed by general questionnaires applied in both groups and by specific instruments restricted to multiple sclerosis. Multiple regression models were applied to assess relationships between predictors and outcome.

**Results:**

age, motor dysfunction and neuropsychiatric symptoms explained 56.6% of quality of life of the multiple sclerosis group. Age and neuropsychiatric symptoms explained 36.6% of quality of life in the control group. Age impacted more the multiple sclerosis group than the control group. Neuropsychiatric symptoms affected both groups similarly. Motor dysfunction impacted 21.9% of the quality of life in multiple sclerosis.

**Conclusions:**

the predictors explained considerable variance of quality of life in multiple sclerosis, which should guide public health policies.

## RESUMO

**Objetivos:**

investigar o impacto da idade, da disfunção motora e dos sintomas neuropsiquiátricos sobre a qualidade de vida de pessoas com esclerose múltipla na comparação com controles saudáveis.

**Métodos:**

141 participantes foram testados em uma única sessão. As avaliações foram compostas por questionários gerais aplicados em ambos os grupos e por instrumentos específicos à esclerose múltipla. Modelos de regressão múltipla foram usados para avaliar relações entre preditores e desfecho.

**Resultados:**

idade, disfunção motora e sintomas neuropsiquiátricos explicaram 56,6% da qualidade de vida do grupo esclerose múltipla. Idade e sintomas neuropsiquiátricos corresponderam a 36,6% da qualidade de vida do grupo-controle. Idade impactou mais o grupo esclerose múltipla do que o grupo-controle. Sintomas neuropsiquiátricos afetaram os grupos semelhantemente. A disfunção motora impactou 21,9% da qualidade de vida na esclerose múltipla.

**Conclusões:**

os preditores explicaram considerável variação da qualidade de vida na esclerose múltipla, o que deve nortear políticas públicas de saúde.

## RESUMEN

**Objetivos:**

investigar el impacto de edad, disfunción motora y síntomas neuropsiquiátricos en la calidad de vida de personas con esclerosis múltiple en comparación con controles saludables.

**Métodos:**

141 participantes fueron evaluados en una sesión. Las evaluaciones fueron compuestas por cuestionarios generales aplicados en ambos grupos y por instrumentos específicos a la esclerosis múltiple. Modelos de regresión múltiple fueron utilizados para evaluar relaciones entre predictores y resultado.

**Resultados:**

edad, disfunción motora y síntomas neuropsiquiátricos explicaron 56,6% de la calidad de vida en la esclerosis múltiple. Edad y síntomas neuropsiquiátricos explicaron 36,6% de la calidad de vida del grupo control. Edad afectó más la esclerosis múltiple que al grupo de control. Síntomas neuropsiquiátricos afectaron los grupos similarmente. La disfunción motora impactó 21,9% de la calidad de vida en la esclerosis múltiple.

**Conclusiones:**

los predictores explicaron una considerable variación de la calidad de vida en la esclerosis múltiple, lo que debe guiar políticas de salud pública.

## INTRODUCTION

Multiple sclerosis (MS) is the most prevalent demyelinating disease that affects the central nervous system. The disease is characterized by immune-mediated inflammation and axonal degeneration that impact the motor, sensitive and autonomic systems^(1-3)^.

Physical decline is common in MS^(4-6)^. With the progression of the disease, physical decline causes social isolation and affects patient’s health-related quality of life^(7-8)^.

Quantifying the impact of MS is one of the most important determinants for optimizing the care and improving quality of life^(8-9)^. In some cases, however, the needs of patients do not come through with the goals stipulated by the health care team^(10-11)^. This usually happens when psycho-social factors do not receive the necessary attention^(12-13)^.

Divergences between patients and health care professionals cause problems in the performance of a good clinical approach, which may affect patient’s confidence as well as the therapeutic procedures to follow^(14-15)^. Seeking to explore this question, researchers performed an in-depth analysis about the impact of non-conventional factors on quality of life in individuals with MS.

The interference of the motor dysfunction is well documented in the literature^(16)^. The impact of neuropsychiatric symptoms is presented by some studies^(13,17)^. The data, however, is still inconclusive when comparing the interference that such factors have in subjects with and without MS.

Authors believe that this study should be of interest of readers of the Brazilian Journal of Nursing (*Revista Brasileira de Enfermagem*), as it might help improving the role of health care professionals on the care systematization of MS patients^(18-20)^.

This research was designed with the prospect of contributing to improving reflection among the relationship between the predictors and outcomes. The main hypothesis was that people with MS have a decrease health-related quality of life compared to control peers, and that age, motor dysfunction and neuropsychiatric symptoms have a higher interference on MS due to an associative-effect with disease severity.

## OBJECTIVES

To analyze the quality of life of subjects with MS and healthy control peers, and to investigate how age, motor dysfunction and neuropsychiatric symptoms impact such outcome.

## METHODS

### Ethical aspects

This research was approved by the Ethics Committee of the Federal University of Mato Grosso do Sul. All participants provided written consent prior to the assessments.

### Design, setting and period of study

This is a cross-sectional design study made up of two independent groups: MS and control. The research was conducted at Federal University of Mato Grosso do Sul in the years 2019 and 2020. The methodological procedures were reported according to the STROBE statement checklist.

Participants with MS were recruited at the Neurologic Outpatient Clinic of the Hospital Maria Aparecida Pedrossian - an institution considered a reference in the treatment of MS. Control peers were select in the community. Authors randomly searched for participants of the control group, considering similar anthropometric and socio-demographic characteristics of patients included in the MS group.

### Sample studied

The sample size calculation involved the delimitation of the alfa in 5%, the statistical power in 80%, and the effect size in 0.46^(21)^. The analysis suggested a minimum of 120 participants, 60 in the MS and 60 in the control group. This survey included 141 participants and ended up with a sample size 16.6% higher than the minimal stipulated by previous analysis.

Inclusion criteria involved community dwelling subjects with relapsing remitting MS, all sedentary, aged 18 years or above at entry, and with disease severity between zero to six according the Expanded Disability Status Scale^(22)^. The exclusion criteria involved participants with neurological conditions other than MS, history or in use of psychotropic or antipsychotic drug, and subjects with cognitive decline.

Healthy control peers were included to compare predictors of quality of life in subjects with and without MS. The selection criteria of the control group matched with anthropometric and socio-demographic characteristics of the MS group.

### Methodological procedures

The assessments of this study involved one main outcome (quality of life) and three predictors: age, motor dysfunction and neuropsychiatric symptoms. The Short-Form Health Survey 36 (SF-36)^(23)^ was used to assess participants’ quality of life. This self-administrated questionnaire is widely used for measuring individuals’ perception of different healthy domains. Each domain is standardized so that scores range from zero to 100 where higher scores represent better quality of life. Authors opted to use this instrument because of its capability to measure quality of life in different populations, being adequate for MS and healthy subjects^(24-25)^.

The assessment of motor dysfunction was restricted to the MS group. This variable was evaluated by disease duration (defined as time since diagnosis) and by Expanded Disability Status Scale score^(22)^. This score consists of an ordinal rating system ranging from zero (normal neurological exam) to ten (death due to MS). The chief neurologist of the Neurologic Outpatient Center was responsible for evaluating participants’ scores. The authors opted to use this questionnaire because of its suitability to detect patient-relevant endpoints in MS^(26)^.

The Hospital Anxiety and Depression scale (HADS)^(27)^ is a screening tool that was designed to assess the levels of anxiety and depression in a non-psychiatric population attending medical clinics. It is comprised of 14 questions divided into two sections: seven questions are related to anxiety and the other seven are focused on depression. The higher the score it was, the higher the level of anxiety and depression of the evaluated person would be. HADS was included because it has a high criterion-related validity for depression and anxiety in MS and in healthy subjects^(28-29)^.

The Mini Mental State Examination (MMSE)^(30)^ was included to evaluate general cognition of the participants. As cognitive dysfunctions are common in MS^(31-32)^, authors opted to assess participants’ cognitive scores as a way of controlling possible interference of cognitive decline on the results. In this study, cognition was used as exclusion criteria. Normal parameters on the MMSE was used according to recommendations provided by Brucki and colleagues^(33)^.

### Data analysis

Data analyses involved descriptive and inferential statistics. As the parametric precepts were not contemplated on all variables, authors used non-parametric statistics as a standardize procedure.

The characterization of the groups was done by median and interquartile range. The use of median and interquartile range is adequate for substitution of mean and standard deviation when parametric precepts are not contemplated^(34)^.

The between-group analyses were assessed with the chi-squared test and with the Mann Whitney U-test. In order to investigate how the predictors affected quality of life in each group, a multiple regression analyses was performed with the addition of three independent blocks of measures: age, motor dysfunction (restricted to the MS group) and neuropsychiatric symptoms. The procedure is described in R^2^. The level of significance was set at 5%.

## RESULTS

Participants’ recruitment was initiated with the MS group. Ninety patients with MS were originally selected. Due to eligibility criteria, twenty participants were excluded of the study. Reasons for exclusion were cognitive decline (n=14), participants under the age of 18 (n=3) and refusal to participate in the research (n=3). After including 70 patients with MS, the control group was recruited taking the anthropometric and sociodemographic aspects of the MS group in consideration. In the control group, eighty participants were invited into the study, but nine subjects refused to participate due to personal reasons. The final sample size of the control group was formed by 71 participants.

Data from table 1 shows that both groups were homogenous for sample size (p=0.933), sex (p=0.956), age (p=0.439), cognition (p=0.079), level of anxiety (p=0.950) and level of depression (p=0.446). The MS group had a disease duration of four years and disease severity of 2.5 points in the Expanded Disability Status Scale.

**Table 1:** Anthropometry and clinical predictors in the Multiple Sclerosis and control groups, Campo Grande, Mato Grosso do Sul, Brazil, 2020

Variables	MS group	Control group	p
Sample size, n	70	71	0.933
Sex, Male - Female	21 - 49	21 - 50	0.956
Age, Years	37.0 (19.2)	38.0 (18.0)	0.439
Mini-Mental State Examination, score	28.0 (2.0)	29.0 (2.0)	0.079
Level of anxiety, score	7.0 (6.0)	7.0 (5.0)	0.950
Level of depression, score	4.0 (5.2)	4.0 (5.0)	0.446
Disease duration, Years	4.0 (7.0)	-----	-----
Expanded Disability Status Scale, score	2.5 (2.0)	-----	-----

*Data is expressed in median (interquartile range), and frequency of events; P value of the chi-squared test for sample size and sex; P value of the Mann Whitney U-test for the other variables.*

### Quality of life

Quality of life was assessed with the SF-36 questionnaire. Patients with MS presented a decline on quality of life in several dimensions, including physical functioning (p=0.001), role physical (p=0.001), general health (p=0.001), vitality (p=0.002), social functioning (p=0.024) and mental health (p=0.001). Differently, quality of life was similar between MS and control peers for bodily pain (p=0.213) and role emotional (p=0.073). Table 2 detail quality of life of participants with and without MS.

**Table 2:** Scores of quality of life in multiple sclerosis and control groups, Campo Grande, Mato Grosso do Sul, Brazil, 2020

Outcomes	MS group	Control group	P
Physical functioning, score	55.0 (60.0)	90.0 (10.0)	0.001
Role physical, score	50.0 (100.0)	100.0 (25.0)	0.001
Bodily pain, score	62.0 (59.2)	72.0 (23.0)	0.213
General health, score	56.0 (40.0)	82.0 (25.0)	0.001
Vitality, score	60.0 (36.2)	70.0 (20.0)	0.002
Social functioning, score	75.0 (50.0)	100.0 (37.5)	0.024
Role emotional, score	100.0 (75.0)	100.0 (33.3)	0.073
Mental health, score	60.0 (28.0)	76.0 (24.0)	0.001

*Data is expressed in median (interquartile range); P value of the Mann Whitney U-test for all variables.*

### Outcomes and predictors based on the multiple regression model

Regression analyses showed considerable impact of age, motor dysfunction and neuropsychiatric symptoms in participants of the MS and control groups.

The statistical analyses indicated that 56.6% of variability of quality of life in subjects with MS are explained by age, motor dysfunction, and neuropsychiatric symptoms. In control group, 36.6% of the variability of quality of life are explained by age and neuropsychiatric symptoms.

Age impacted up to 19.9% of the quality of life on the MS group and up to 2.9% of the quality of life in the control group. Motor dysfunctions, assessed only in the MS group, impacted mostly the mental health and vitality domains. Neuropsychiatric symptoms impacted similarly the quality of life of both groups. Table 3 shows regression coefficients of predictors and outcome.

**Table 3:** Regression coefficients of the predictors entered in the final model for each outcome measure, Campo Grande, Mato Grosso do Sul, Brazil, 2020

Groups	Predictors	Outcome (R^2^, %)							
		PF	RP	BP	GH	V	SF	RE	MH
Multiple sclerosis	Age	19.9	1.1	5.8	2.5	4.3	3.8	0.3	1.2
	Motor dysfunction	19.8	12.3	9.9	5.4	11.2	19.4	21.9	16.7
	Neuropsychiatric symptoms	10.0	7.3	19.3	22.9	24.2	17.3	9.1	38.7
	Total	49.7	20.7	35.0	30.8	39.7	40.5	31.3	56.6
Control	Age	1.8	1.0	2.9	2.9	0.1	0.1	0.1	0.1
	Neuropsychiatric symptoms	10.5	22.1	19.1	19.0	26.1	19.0	7.7	36.5
	Total	12.3	23.1	22.0	21.8	26.2	19.1	7.8	36.6

Data is expressed in percentage; PF - Physical functioning; RP - Role physical; BP - Bodily pain; GH - General health; V - Vitality; SF - Social functioning; Role emotional; MH - Mental health.

## DISCUSSION

The findings of this study showed that patients with MS have a worse health-related quality of life than control peers. Neuropsychiatric symptoms were the predictors that most impacted the quality of life of subjects with MS, followed by motor dysfunction and age. Age and neuropsychiatric symptoms impacted the quality of life of healthy peers, as well. The understanding of these factors is important to assess patients’ quality of life and to guide the proposal of new public health policies.

The results showed that changes provided by motor dysfunction in MS affected subjects’ emotional, physical, social, and mental functions. Such finding is important because it proves that disease severity impacts not only the physical aspects of the patients but it also plays a role on social and mental domains. This study corroborates previous researches when report motor dysfunction as a predictor of quality of life in MS^(35-39)^. It indicates, furthermore, that in MS the symptoms many times overlap and patients’ treatment becomes challenging^(40-41)^.

Regarding the impact of neuropsychiatric symptoms on patients’ health-related quality of life, this study showed at first that both MS and control groups present similar scores on HADS. Second, it showed that anxiety and depression were associated mainly with mental health and vitality. The similar values of neuropsychiatric symptoms in subjects with and without MS went against authors’ original hypothesis, as it was expected that neuropsychiatric symptoms would play a bigger interference in the MS group.

Authors highlight two explanations for the neuropsychiatric pattern seen in the MS and control group. First, the Expanded Disability Status Scale showed that this sample was formed by individuals with MS in the mild to moderate stages of the disease. It is possible that patients with advanced MS would present more neuropsychiatric symptoms than subjects in the initial stages. Second, others neuropsychiatric factors not analyzed in this study could be affecting patients’ health on a bigger extend, such as apathy, aggression, sleep disturbances, confusion and agitation^(41-42)^. Confirmation of these hypotheses requires further studies.

Age has impacted the quality of life of all subjects, especially in the MS group. Considering that both groups were formed by young adults, the bigger interference of age in the MS group was surprising. In fact, the quality of life scores seen in the MS group was similar in many aspects to the quality of life seen in older adults with mobility problems^(43)^. This result is interesting and it can be justified to the fact that much of the symptoms seen in MS are common to aging, but early^(44-45)^. The impact of ~20% of age on physical function supports this finding. It shows, furthermore, that individuals with MS need to manage simultaneously with normal aging process and with the disability related to the disease.

Cognitive dysfunctions are common in MS and they are responsible for impacting patients’ health-related quality of life^(46)^. In spite of that, the authors decided for excluding subjects with cognitive decline because of its potential in affecting subjects’ comprehension on the instruments. On one hand, this exclusion caused a significant percentage of sample loss (n=14; ~15% of the MS group). On the other hand, excluding subjects with cognitive decline gave the authors the certain that the results are reliable to what the participant was feeling.

At last, it is important to highlight that only sedentary participants were included in this study. Authors opted to restrict the sample to such profile because physical activity has been proven to be beneficial in MS, impacting subjects’ quality of life^(47)^.

### Study limitations

Authors recognize two main limitations. First, the results are restricted to mild-moderate stage subjects with MS that does not present cognitive impairments. Second, the regression models could not explain 43.7% of the variability of quality of life in subjects with MS. This should encourage new studies seeking to investigate the impact of other predictors on the quality of life in people with MS.

### Contributions to the area

This study highlighted important topics about which health care professionals should be aware before assisting subjects with MS. The results provided new information about the impact of age, motor dysfunction and neuropsychiatric symptoms on the quality of life of people with MS - which may be of interest of readers of the Brazilian Journal of Nursing.

## CONCLUSIONS

This study showed that age, motor dysfunction and neuropsychiatric symptoms impact the quality of life of subjects with MS.

The greater influence that neuropsychiatric symptoms and age had upon the results suggest that treating disease alone might not be as effective to improve the quality of life as making a global assistance to the patient.

The findings support the development of new therapies performed by multi-professional teams to control not only the progression of the disease but also to create stimulus helping patients on several health domains. This approach provides guidance to ensure that people with MS are well assisted.
